# Clival Chordoma: A Rare Finding in Children

**DOI:** 10.5334/jbsr.2334

**Published:** 2021-02-22

**Authors:** Manon Vanhamel, Vincent VandeVyver, Koenraad Verstraete

**Affiliations:** 1 University of Ghent, BE; 2 AZ Alma, BE; 3 Academic Chief Department of Radiology Ghent University Hospital and Ghent University, BE

**Keywords:** Chordoma, Pediatrics, Malignant, Tumor, Notochord, Clivus

## Abstract

**Teaching point:** Clival chordoma is a rare find in children, presenting as a locally destructive, T2 hyperintense, and strongly enhancing mass.

## Case study

**Figure 1:**
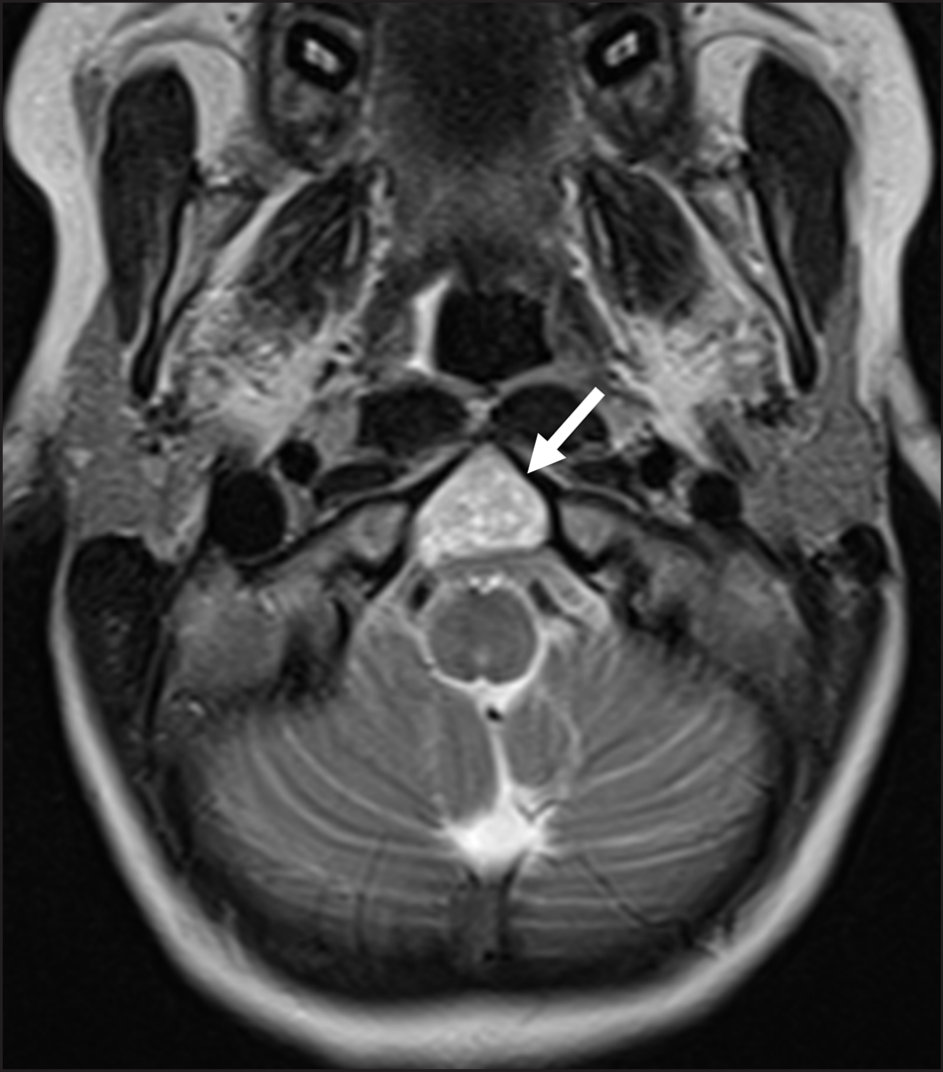


**Figure 2:**
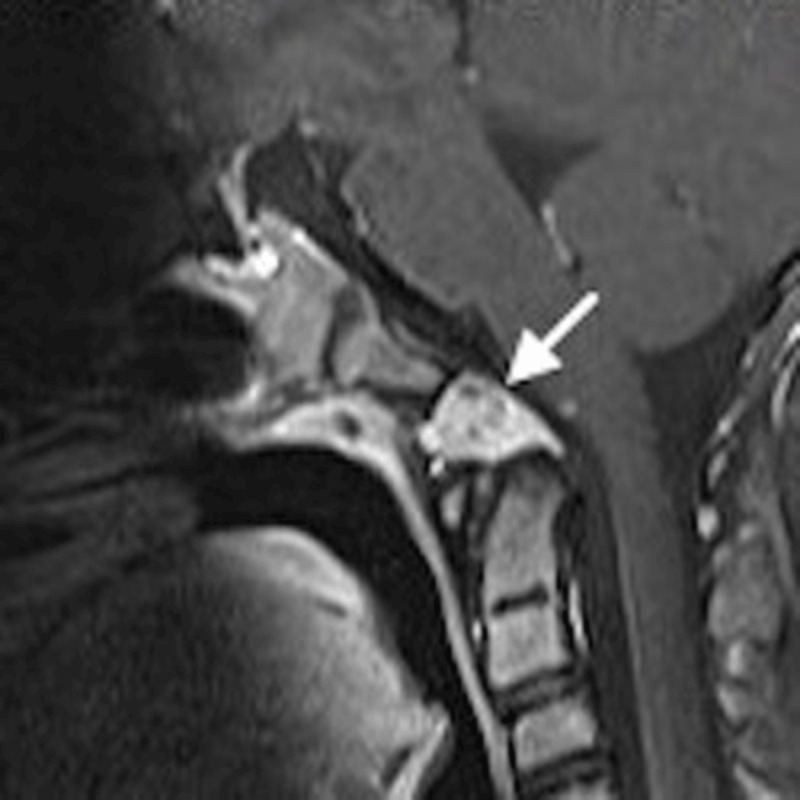


**Figure 3:**
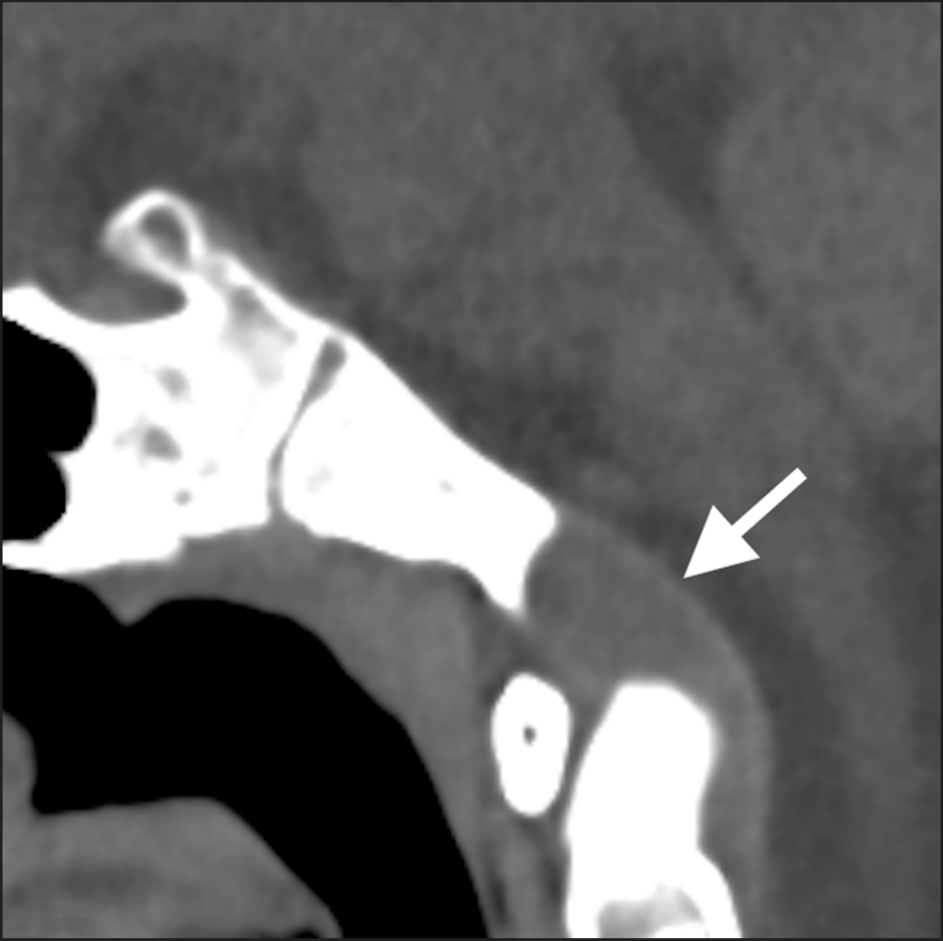


An eleven-year-old girl was referred to a pediatrician because of a persistent headache. Physical examination was normal. Magnetic resonance imaging (MRI) of the brain showed a 2 cm sharply delineated mass in the caudal part of the clivus, at the height of the basion, with low signal-intensity on T1-weighted imaging. On T2-weighted imaging, the lesion appeared heterogeneously hyperintense (***Figure 1***, arrow), without any signal suppression on the fluid-attenuated inversion recovery (FLAIR) sequence. There was a strong heterogenous contrast enhancement (***Figure 2***, arrow). Computed tomography (CT) showed a sharply delineated soft-tissue mass, with neither calcification nor bone tissue (***Figure 3***, arrow). No other osteolytic bone lesion was detected. Based on imaging findings, the diagnosis of chordoma was proposed. The lesion was resected surgically and the diagnosis of chordoma was confirmed on histology. Postoperatively, the patient underwent several sessions of radiation therapy and is currently under follow-up, free of relapse.

## Comment

Chordoma is a slow-growing, malignant tumor, arising from cellular remnants of the primitive notochord. Chordomas have an incidence rate of less than 1 per 1,000,000 population, with a peak incidence between the fourth and sixth decades. It is a rare condition in children; less than 5% occur under 20 years, which makes this eleven-year-old girl an exceptional case. The most common location of the tumor in adults is sacrococcygeal, while in children, the craniovertebral junction is the most common site (54%). The tumor is mainly locally destructive, and distant metastases are relatively rare. The symptoms of a clival chordoma include diplopia and headache. The tumor presents as a lobulated, gray mass, with possible areas of calcification and hemorrhage. On CT it appears as a sharply defined, expansile, soft tissue mass, with extensive lytic bone destruction. On MRI the tumor is typically hyperintense to the cerebrospinal fluid on a T2-weighted sequence and may have low-signal fibrous septations. On a T1-weighted sequence, the tumor appears heterogeneously hypo- to isointense compared to the bone marrow, showing mild to intense enhancement after administration of contrast. Differential diagnosis should be made with benign notochordal cell tumors, chondrosarcomas, and Langerhans cell histiocytosis. Benign notochordal cell tumors usually have no contrast enhancement. The majority of chondrosarcomas are located off the midline, have a lytic aspect with calcifications, and show a serpiginous enhancement. Langerhans cell histiocytosis appears as lytic punched out lesions, typically in the skull, long bones, and mandible and shows a diffuse contrast enhancement. A chordoma is usually treated by surgical resection with adjuvant radiation therapy. After successful treatment, recurrence is not uncommon, especially along the operative tract. The five-year overall survival rate of pediatric chordomas in the literature varies between 60–80%. While the prognosis in children is better than in adults, patients younger than 5 years old generally present with very aggressive chordomas and poor outcomes [1].
